# Endoscopic submucosal dissection of papillary adenocarcinoma of stomach; protocol for a systematic review and meta-analysis

**DOI:** 10.1097/MD.0000000000013905

**Published:** 2018-12-28

**Authors:** Chang Seok Bang, Jae Ho Choi, Jae Jun Lee, Gwang Ho Baik

**Affiliations:** a Department of Internal Medicine; b Institute of New Frontier Research; c Department of Anesthesiology and Pain Medicine, Hallym University College of Medicine, Chuncheon, Korea.

**Keywords:** early gastric cancer, endoscopic submucosal dissection, papillary adenocarcinoma

## Abstract

**Background:**

Endoscopic submucosal dissection (ESD) is a primary treatment for the early gastric cancer (EGC) who has a negligible risk of lymph node metastasis. Papillary adenocarcinoma (PAC) of stomach is a rare histologic variant of gastric cancer and categorized into EGC with differentiated-histology. However, aggressive features such as higher rate of lymphovascular invasion (LVI) or submucosal invasion have been reported, whereas comparable lymph node metastasis (LNM) rate to the lesions meeting the current ESD criteria also has been reported. This study aimed to evaluate the feasibility of ESD for EGC with PAC.

**Methods:**

We will search the core databases (MEDLINE (through PubMed), the Cochrane Library, and Embase) from their inception to December 2018 by 2 independent evaluators. The P.I.C.O. is as follows; Patients: who have EGC with PAC, Intervention: ESD or surgery, Comparison: none, Outcome: at least one among the rate of complete resection, curative resection, en bloc resection, recurrence, procedure-related adverse event, LVI or LNM that enabled an evaluation of the feasibility of ESD. All types of study design with full text will be sought and included. The risk of bias will be assessed using the ROBINS-I tool. Descriptive data synthesis is planned, and quantitative synthesis will be used if the included studies are sufficiently homogenous. Publication bias will be assessed with quantitative analyses if more than 10 articles are enrolled.

**Results:**

The results will provide evidence for efficacy and safety of ESD for EGC with PAC.

**Conclusion:**

This study will provide evidence of ESD for EGC with PAC.

## 1 Introduction

Endoscopic submucosal dissection (ESD) is a primary treatment of early gastric cancer (EGC) for the lesions satisfying specific indications implying negligible risk of lymph node metastasis (LNM).^[1]^ This enables an en bloc resection and organ preservation, thereby avoiding invasive surgery. These indications are histologically categorized by EGC with differentiated- (EGC-DH) and undifferentiated-type histology (EGC-UH) and have specific size, morphologic, and histologic conditions.^[1,2]^

The absolute indications for ESD of EGC include EGC-DH of less than 2 cm in the absence of ulceration and lymphovascular invasion (LVI).^[2]^ This indication for ESD has been expanded with advances in endoscopic skills and expertise, and these expanded indications include mucosal EGC-DH without ulceration irrespective of tumor size; mucosal EGC-DH with ulceration measuring less than 3 cm; mucosal EGC-UH measuring less than 2 cm without ulceration; EGC-DH with minute submucosal invasion (≤500 μm, SM1) measuring less than 3 cm, without evidence of LVI.^[3–5]^

Among the 5 main histologic types (tubular, papillary, mucinous, poorly cohesive, and mixed) of gastric adenocarcinoma in the World Health Organization classification, papillary adenocarcinoma (PAC) of stomach is a rare variant and it is histologically characterized by finger-like papillary epithelial processes lined with columnar neoplastic cells with a central fibrovascular core.^[6,7]^ It is categorized into EGC-DH; however, aggressive features such as higher LVI or submucosal invasion rate have been reported,^[8,9]^ whereas comparable LNM rate to the lesions meeting the current ESD criteria also has been reported.^[10]^ Moreover, therapeutic outcomes of ESD for EGC with PAC has not been clearly described. This study aimed to evaluate the feasibility of ESD for EGC with PAC.

## 2 Methods

This systematic review and meta-analysis study will fully adhere to the principles of the Preferred Reporting Items for Systematic reviews and Meta-Analyses (PRISMA-P) checklist.^[11]^ This study was registered at PROSPERO (https://www.crd.york.ac.uk/prospero) on November 2018 (registration number, CRD42018115575) before the study was initiated. The approval of institutional review board was exempted due to the characteristics of this study (collecting and synthesizing data from published studies).

### 2.1 Literature searching strategy

MEDLINE (through PubMed), the Cochrane library, and Embase will be searched using common keywords associated with ESD for EGC with PAC (from inception to December 2018) by 2 independent evaluators (C.S.B., and J.H.C). Medical Subject Heading or Emtree keywords will be selected for searching electronic databases. The abstracts of all identified studies will be reviewed to exclude irrelevant publications. Full-text reviews will be performed to determine whether the inclusion criteria are satisfied in the remaining studies, and the bibliographies of relevant articles will be rigorously reviewed to identify additional studies. Disagreements between the evaluators will be resolved by discussion or consultation with a third evaluator (G.H.B.). The detailed searching strategy is described in Table 1.

**Table 1:**
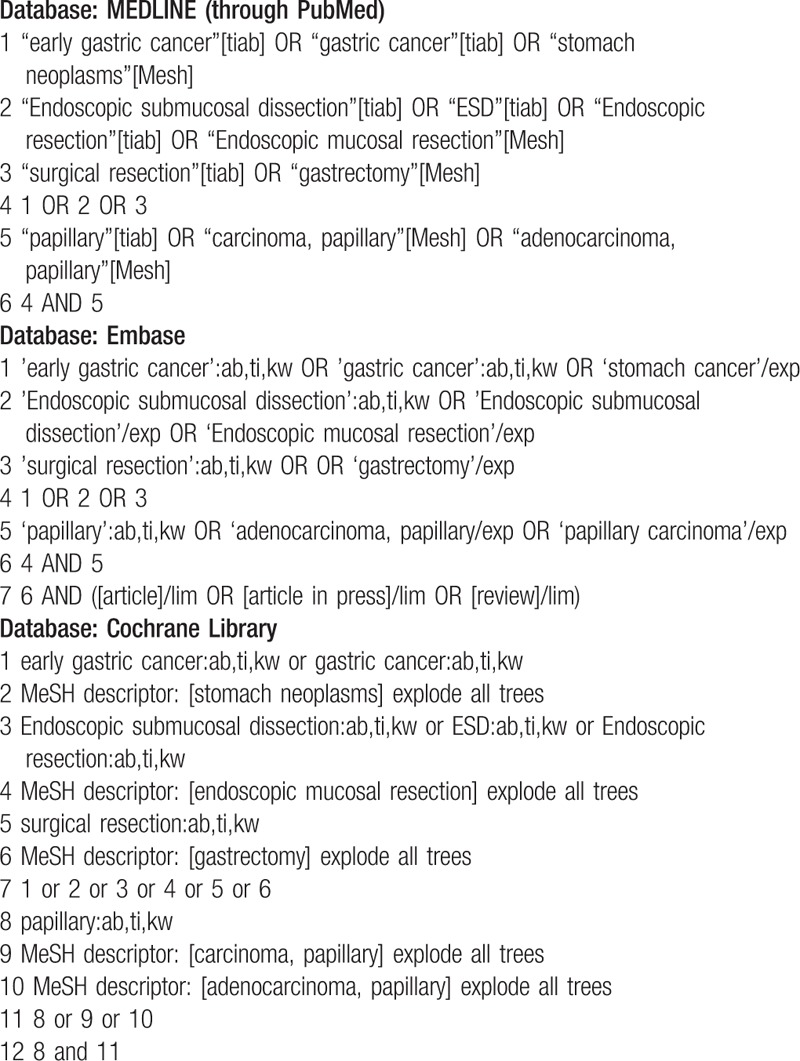
Searching strategy to find the relevant articles.

### 2.2 Selection criteria

We will include studies that met the following criteria: 1. Patients: who have EGC with PAC; 2. Intervention: ESD or other types of endoscopic resection (i.e., endoscopic mucosal resection), preferentially; however, surgical outcomes will be also sought if paucity data exist for endoscopic resection; 3. Comparison: none; 4. Outcome: at least one among the rate of endoscopic therapeutic outcomes such as complete resection, curative resection, en bloc resection, recurrence or procedure-related adverse event, LVI that enabled an evaluation of feasibility of ESD; rate of LNM will be described if paucity data exist for endoscopic resection; 5. Study design: all types including randomized, prospective, retrospective studies or case studies; 6. Studies of human subjects; and 7. Full-text publications. Studies that met all of the inclusion criteria will be sought and selected. The exclusion criteria are as follows: 1. review articles; 2. guidelines, consensus documents or expert position papers; 3. comments, letters, brief reports, proceedings, or protocol studies; 4. publications with incomplete data; and 5. meta-analysis articles. Studies meeting at least 1 of the exclusion criteria will be excluded from this analysis. The language of publication will not be restricted.

### 2.3 Methodological quality

The methodological quality of the included publications will be assessed using the Risk Of Bias In Non-randomized Studies-of Interventions (ROBINS-I) tool.^[12]^ The ROBINS-I tool contains 7 domains, including “bias due to confounding” and “bias in selection of participants into the study” at preintervention, “bias in classification of intervention” at intervention and “bias due to deviations from intended interventions”, “bias due to missing data,” “bias in measurement outcomes,” and “bias in selection of the reported result” at postintervention.^[12]^ Each domain is determined to exhibit low-, moderate-, serious-, or critical risk of bias. No information category will be used only when insufficient data are reported to permit a judgment.^[12]^ The overall risk of bias judgment is determined based on the interpretation of each domain level, and low risk indicates that the study is comparable to a well-performed randomized trial for all domains being evaluated. Moderate risk of bias indicates that the evidence of study is sound for a nonrandomized study but not comparable to a randomized trial (low or moderate risk of bias for all domains). Serious risk of bias indicates the presence of important problems (serious risk of bias in at least 1 domain, but not at critical risk of bias in any domain). Critical risk of bias indicates that the study is problematic to provide any useful evidence (critical risk of bias in at least 1 domain).^[12]^

Two of the evaluators (C.S.B. and J.H.C.) will independently assess the methodological qualities of all the included studies, and any disagreements between the evaluators will be resolved by discussion or consultation with a third evaluator (G.H.B.).

### 2.4 Primary and modifier-based analyses

Two evaluators (C.S.B. and J.H.C.) will independently use the same data fill-in form to collect the primary summary outcome and modifiers in each study, and disagreements between the 2 evaluators will be resolved by discussion or consultation with a third author (G.H.B). The definition of primary therapeutic outcome is as follows: en bloc resection is defined as complete removal of cancer in a single piece without fragmentation. Complete resection is defined as removal of cancer with no neoplastic components at the lateral or vertical margins and without LVI on microscopic examination. Curative resection is defined as (1) removal of cancer with 20 mm or smaller intramucosal lesions without ulceration (scar), neoplastic components at the lateral or vertical margins, and LVI for EGC-UH; (2) removal of cancer with 20 mm or smaller intramucosal lesions without ulceration; (3) removal of cancer with 20 mm or larger intramucosal lesions without ulceration (scar); (4) removal of cancer with less than 30 mm intramucosal lesions with ulceration (scar); (5) removal of cancer with less than 30 mm submucosal invasion depth of <500 μm, without ulceration (scar), neoplastic components at the lateral or vertical margins, and LVI for EGC-DH.^[1,2,4]^ If the lesion does not meet these curative criteria, it is regarded as noncurative resection.^[1,2,4]^ Recurrence is defined as reappeared at the site of the lesion (local recurrence) or synchronous, metachronous or distant metastatic lesions, and adverse event of ESD is defined as the cancers whose treatment resulted in procedure-related gastric hemorrhage or perforation.^[3]^

Narrative (descriptive) synthesis is planned, and quantitative synthesis will be used if the included studies are sufficiently homogenous. Authors previously reported therapeutic outcomes of ESD for EGC-UH using pooled meta-analysis of crude outcomes of each study.^[13]^ The common effect size will be extracted from each study using method previously described (pooled meta-analysis of crude outcomes)^[13]^, and we will also perform sensitivity and meta-regression analyses using the modifiers identified during the systematic review to confirm the robustness of the main result and to identify the reason of heterogeneity. If paucity data exist for endoscopic resection of EGC with PAC, the rate of LNM will be described and retrospective application of ESD criteria for surgically resected specimen will be performed, whenever possible.

### 2.5 Statistical analysis

Comprehensive Meta-Analysis Software (version 3, Biostat; Borenstein M, Hedges L, Higgins J and Rothstein H. Englewood, NJ, USA) and R version 3.2.3^[14]^ will be used for this meta-analysis. We will calculate the pooled rate of an en bloc resection, complete resection, curative resection, recurrence and adverse event rates divided by gastric hemorrhage and perforation, LVI or LNM, whenever possible. If paucity data exist, pooled rate of LNM will be described. Heterogeneity will be determined using the *I*^*2*^ test developed by Higgins, which measures the percentage of total variation across studies.^[15]^*I*^*2*^ will be calculated as follows: *I*^*2*^ (%) = 100 × (*Q*-d*f*) / *Q*, where *Q* is Cochrane's heterogeneity statistic, and d*f* signifies the degrees of freedom. Negative values for *I*^*2*^ will be set to zero, and an *I*^*2*^ value over 50% was considered to be of substantial heterogeneity (range: 0–100%).^[16]^ Pooled-effect sizes with 95% confidence intervals (CIs) will be calculated using the DerSimonian and Laird random effects model meta-analysis, and sensitivity analyses will be performed using the Mantel–Haenszel fixed-effect model meta-analysis.^[17]^ These results will be confirmed by the *I*^*2*^ test. Significance will be set at *P* = .05. Publication bias will be evaluated using Begg's funnel plot, Egger's test of the intercept, Duval and Tweedie's trim and fill, and Begg and Mazumdar's rank correlation test.^[18–22]^

## 3 Discussion

This is the protocol of a systematic review and meta-analysis for the therapeutic outcomes of ESD for EGC-PAC. PAC is known to be rare histologic type approximately accounting for 6% to 11% of all gastric cancers and 1% to 18% of EGCs from previous reports.^[8–10,23–26]^ Because of its rarity in incidence, the characteristic of clinicopathologic features and therapeutic outcomes have not been clearly established.

PAC is generally defined as a tumor in which more than 50% of the involved area contains papillary structures across studies.^[8–10]^ Although PAC is categorized into the differentiated group in the Japanese classification,^[5]^ PAC mixed with other differentiated-type EGC, or PAC mixed with undifferentiated-type EGC, is also classified according to the predominant component in the entire cancer. Surgical data on EGCs with PAC showed an LNM rate of 17.9% for the all lesions, an LNM rate of 11.8%, and an LVI rate of 17.6% for lesions that met the current ESD criteria, indicating adoption of the same ESD criteria for EGC-DH is unlikely.^[9]^ Retrospective analysis of endoscopically resected EGCs with PAC also showed that the presence of papillary structure was an independent risk factor for lymphatic involvement (odds ratio 8.1, 95% confidence interval: 3.2–20.6).^[27]^ However, another study with surgical data on EGC with PAC showed LNM rates comparable to those in differentiated tubular adenocarcinoma (1.5%, 1.1%, and 4.0% for mucosal EGCs and 9.45%, 11.9%, and 17.6% for submucosal EGCs, in EGC with PAC, differentiated tubular EGC, or EGC-UH, respectively), despite persistent aggressive features of higher LVI or submucosal invasion rates.^[10]^ Moreover, no LNM occurred in lesions that met the current ESD criteria for PAC.^[10]^ In terms of the long-term outcomes, there was 5.2% of metachronous recurrence; however, no extra-gastric recurrences in patients who achieved curative resection for EGC with PAC during median follow-up of 58 months.^[28]^

To explain this discrepancy between studies, further research is needed and the results of this study will provide evidence for validity of current ESD criteria in addition to the technical feasibility of ESD for EGC-PAC.

## Author contributions

**Conceptualization:** Chang Seok Bang.

**Data curation:** Chang Seok Bang, Jae Ho Choi, Jae Jun Lee, Gwang Ho Baik.

**Formal analysis:** Chang Seok Bang, Jae Ho Choi, Jae Jun Lee.

**Funding acquisition:** Chang Seok Bang.

**Investigation:** Chang Seok Bang, Jae Ho Choi, Jae Jun Lee, Gwang Ho Baik

**Methodology:** Chang Seok Bang

**Project administration:** Chang Seok Bang

**Resources:** Chang Seok Bang, Jae Ho Choi, Jae Jun Lee, Gwang Ho Baik.

**Visualization:** Chang Seok Bang.

**Writing – original draft:** Chang Seok Bang.

**Writing – review & editing:** Chang Seok Bang.

Chang Seok Bang orcid: 0000-0003-4908-5431.
